# Growth and health outcomes at school age in HIV-exposed, uninfected Zambian children: follow-up of two cohorts studied in infancy

**DOI:** 10.1186/s12887-015-0386-8

**Published:** 2015-06-06

**Authors:** Laura Nicholson, Molly Chisenga, Joshua Siame, Lackson Kasonka, Suzanne Filteau

**Affiliations:** Faculty of Epidemiology and Population Health, London School of Hygiene & Tropical Medicine, Keppel Street, London, UK; Department of Obstetrics and Gynecology, University Teaching Hospital, Lusaka, Zambia

**Keywords:** HIV exposure, Growth, Body composition, School performance

## Abstract

**Background:**

Early growth and health of HIV-exposed, uninfected (HEU) children is poorer than that of their HIV-unexposed, uninfected (HUU) counterparts but there is little information about longer term effects of early HIV exposure. We previously recruited two cohorts of HEU and HUU Zambian infants and documented the poorer infant growth and health of the HEU compared to the HUU children. We followed up HEU and HUU children from these cohorts when they were school-aged and compared their growth, health, biochemical markers of acute or chronic disease, and school grades.

**Methods:**

We recruited 111 HEU and 279 HUU children aged 6–12 years. We measured anthropometry, determined health by questionnaire and clinical examination, viewed the child’s most recent school report, and measured blood pressure, haemoglobin (Hb), HbA1c, glucose, cholesterol, and C-reactive protein (CRP).

**Results:**

Anthropometric measures were lower among HEU than HUU children, significantly so for hip circumference (age- and sex-adjusted difference −1.74 cm; 95 % confidence interval (CI) -3.24, −0.24; *P* = 0.023) and mid-upper-arm circumference (adjusted difference −0.63 cm, 95 % CI −1.23, −0.04; *P* = 0.037) and with borderline effects for body mass index, thigh circumference and subscapular skinfolds. HEU children had significantly lower total, trunk, and limb fat percentages. All anthropometric and body composition differences became non-significant after adjustment for sociodemographic variables which differed between HEU and HUU children. More HEU than HUU children reported minor illnesses and were prescribed medication at the time of visit. There were no differences in biochemical markers between groups. HEU children had lower math grades than HUU children even after adjustment for socioeconomic variables.

**Conclusions:**

Although HEU children were smaller and had lower percent fat than HUU children, this appeared to be due mainly to their poorer socioeconomic status. Reasons for lower school grades require further research.

**Electronic supplementary material:**

The online version of this article (doi:10.1186/s12887-015-0386-8) contains supplementary material, which is available to authorized users.

## Background

With increasing access to antiretroviral therapy (ART) in Africa, including for pregnant women both to prevent mother-to-child HIV transmission and for women’s own health, the number of HIV-exposed, uninfected (HEU) infants is increasing. Any health problems these children experience, even if minor individually, have potential enormous public health impact. There is evidence from Africa that HEU children have lower birth weight, poorer early growth, and poorer health and survival compared with HIV-unexposed children [1–4]. Mechanisms for this are unclear, likely multifactorial, and with different contributions in different populations and at different times. Potential mechanisms include exposure to maternal illness and inflammation [5, 6], exposure to ART drugs [1], increased exposure to other infections, notably cytomegalovirus [7], immune abnormalities beginning early in life and influencing both disease resistance and responsiveness to standard vaccines [8], reduced duration of breastfeeding [9], and sociodemographic factors related in part to ill parents being unable to care optimally for their infants [4]. It is important to note that, in highly HIV-endemic areas of Sub-Saharan Africa, HIV-infected and uninfected women may have largely similar socioeconomic status (SES), racial and demographic profile [10–12], unlike in Europe and North America.

To date there appears to be little information about growth and health of older African HEU children. It is possible that in these children *in utero* insults resulting from HIV exposure have long term effects. The children may be at higher risk of infectious diseases because of their increased risk of stunting which is associated with increased morbidity [13], and their immune abnormalities and altered protection from vaccines [8]. Stunting is also associated with poor cognitive development in HIV-exposed [14, 15] and unexposed children [16]. HEU children may also be at increased risk for obesity and associated chronic diseases because of lower birth weight and early increases in body mass index (BMI) [17] or skinfold thicknesses [2]. Information about the longer term health outcomes of HEU children is essential in order to design programmes to mitigate any problems.

We investigated in a cross-sectional study the health, growth, body composition, biochemical markers of acute or chronic diseases, and school performance of two cohorts of school-aged HEU and HIV-unexposed, uninfected (HUU) Zambian children.

## Methods

### Participants

The children were previous participants in one of two research projects conducted by the team in Chilenje, Lusaka, Zambia. HIV-infected and uninfected mothers of the children in the Breastfeeding and Postpartum Health (BFPH) longitudinal cohort study [11] were recruited when pregnant. BFPH children were born between 2001 and 2004. Detailed information on maternal and infant health, infant feeding, and infant growth was collected until age 16 weeks. HIV status of all mothers was known through antenatal testing at the local government clinic. At the time of the study the only antiretroviral regimen available for prevention of mother-to-child transmission (PMTCT) in the area was perinatal nevirapine to both mother and infant. The median duration of exclusive breastfeeding was 6 weeks for HIV-infected women and 9 weeks for HIV-uninfected women [9] and the median duration of any breastfeeding was 17 months and 19 months for these groups, respectively (unpublished). The only sociodemographic factors which differed between HIV-infected and uninfected women were that the infected women were slightly older and less likely to be primiparous.

Children in the Chilenje Infant Growth, Nutrition and Infection Study (CIGNIS) trial were born between 2005 and 2007. They were recruited at age 6 months and participated until they were 18 months in a randomised controlled trial comparing two locally made complementary foods differing in micronutrient content [10]. At the time of the study perinatal nevirapine was the local regimen for PMTCT. ART was available only for adults with CD4 count < 200 cells/μL until towards the end of the study when the cut-off was changed to < 350 cells/μL; few of the CIGNIS children’s mothers were on any ART. Agreement to HIV-testing of children by antibodies at 18 months, the only test available locally throughout most of the trial, was an inclusion criterion of the study. Children who died or defaulted before 18 months were not tested for HIV. Knowledge of maternal HIV status was not required, although antenatal HIV status from routine government health services was known for 90 % of the women. HIV-infected mothers were older than uninfected mothers, were of lower education and more likely to be in the lowest tertile of an asset index. HIV-infected mothers were less likely to initiate breastfeeding and stopped earlier compared to uninfected mothers [18].

Follow-up for both cohorts of children was March to May 2014, a time chosen based on availability of staff and funding. We used a combination of methods to find the children. First, we remain in touch with some of the mothers through a women’s support group set up originally in Chilenje by mothers in the BFPH study. Second, we tried addresses and mobile phone numbers from the original studies. This was more successful for CIGNIS than BFPH mothers since CIGNIS was more recent and families were thus less likely to have moved or changed phone numbers; in addition, mobile phones were less common at the time of the earlier BFPH study and only wealthier families owned them. Finally, we asked mothers we did find if they were aware of addresses or phone numbers of any other mothers and children from the studies.

### Data collection

Parents and children were invited to Chilenje clinic for a scheduled individual assessment. The visit included demographic, socioeconomic, and morbidity history data by questionnaire and a clinical examination. In addition to general health, outcomes measured focussed on growth and biochemical markers of acute or chronic disease since these are potential concerns among HEU children. Anthropometry (weight, height, mid-upper arm circumference (MUAC), waist, hip and thigh circumferences, triceps and subscapular skinfolds) was measured by an experienced anthropometrist (MC) in triplicate by standard methods [19]; the median was used in analyses. Height and BMI Z scores were calculated using the World Health Organization standards for children aged 5–19 years [20]. Body composition was measured by bioelectrical impedance (Tanita BC418, Chasmors, London, UK) only in children over 7 years since the machine is not designed for younger children. The machine uses internal equations to calculate total and individual limb and trunk lean and fat mass; since fat and lean are calculated by difference, we focussed on percent fat. Blood pressure was measured in all children using a Diamond Mercury B.P. Apparatus (India). Fingerprick blood samples were used for measurement of haemoglobin (Hb), HbA1c, and glucose, all using hand-held instruments from Hemocue (Dronfield, UK). There was a problem with the glucose monitor during part of the study so many results are missing. Venous blood samples were collected in plain tubes for measurement of total cholesterol using a commercial kit on a Pointe 180 analyser (Bactlabs, Nairobi, Kenya) and serum C-reactive protein (CRP), an indicator of systemic inflammation, by commercial ELISA kit (AssayPro, St Charles, MO, USA).

### HIV status and exposure

At follow-up parents were asked whether they or their child had been tested for HIV since the previous BFPH or CIGNIS study. The most recent test result was used to define child HIV status. HIV-infected children had all measurements taken for ethical reasons but were excluded from statistical analyses. Since we were primarily interested in children’s HIV exposure *in utero* or through breastfeeding, children’s HIV exposure was defined by mother’s status during the earlier study. We excluded from analysis children of the 70 CIGNIS mothers of unknown status. Children of HIV-uninfected mothers who had never themselves been tested for HIV were assumed to be HUU. Children of HIV-infected mothers who had not themselves been tested were included as HEU since we expected that by school age most HIV-infected children would show symptoms. The study clinical officer (JS) examined all children and would have referred any children suspected of HIV infection to local services but did not, in fact, find any likely HIV-positive children other than those already known to be positive. We also conducted restricted analyses including as HEU only children confirmed HIV-negative.

### School reports

Mothers were asked to bring their child’s most recent school report to the clinic visit. For some of the younger children the schools did not provide grades, only an indication of how children were performing according to expectation, so these reports were omitted. We took from the reports the children’s grades in English (the language of instruction in Zambian schools) and math/arithmetic as well as the maximum achievable grades in these subjects according to the particular school’s grading system. Grades were expressed as a percent of the maximum achievable.

### Statistical analyses

Data were double-entered into Access databases, cross-checked, cleaned and imported into Stata for analysis. Using principal components analysis [21], an asset index was generated from data on possession of car, bicycle, radio, television, phone, fan and refrigerator plus type of toilet, household water source and whether they owned, rented or shared their accommodation. This index was divided into tertiles of low, middle and high socioeconomic status.

Primary analyses used linear regression to compare HEU and HUU children for all outcomes: anthropometry, total, trunk and limb fat percentage, blood pressure, blood Hb, HbA1c, glucose and CRP, and school grades. These analyses were then adjusted for sociodemographic factors which differed between HUU and HEU children. We also compared baseline characteristics from the original studies which differed between children who were and were not later followed up in order to adjust for these in a missing at random analysis [22].

The sample size was determined pragmatically by the number of children we could find with the limited available time and funds. With the number of children who were available, and given there were about 2 HUU controls per HEU child, we could detect, at 5 % significance, differences in outcomes between groups of about a third of a standard deviation (SD) at 80 % power and 0.4 SD at 90 % power.

### Ethics and consent

The study was approved by the University of Zambia Biomedical Research Ethics Committee and the ethics committee of the London School of Hygiene and Tropical Medicine. Parents provided written informed consent for their children to participate. Children who could not read provided verbal assent and those who could read and write also signed a written assent form; this protocol was considered locally appropriate for school-aged children. Particular care was given to ensure confidentiality of HIV status, including that no child was provided with information about his/her mother’s HIV status.

Children requiring medical intervention were referred to the local government clinic on the same site as the project clinic. HIV-infected children were included in all data collection even though their data were not analysed and they were referred to local HIV services if not already attending these.

## Results

**Figure 1:**
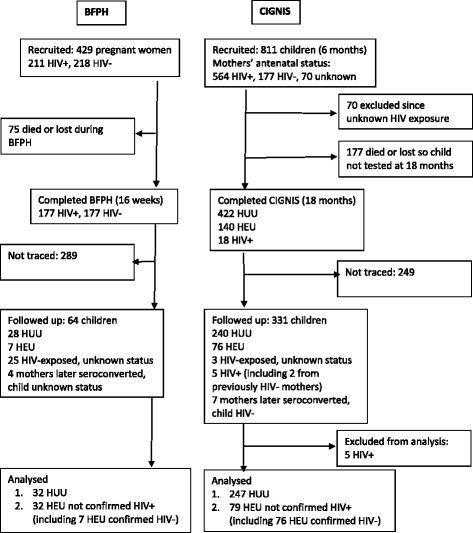
Flow diagram of study participants. BFPH = Breastfeeding and Postpartum Health Study, CIGNIS = Chilenje Infant Growth Nutrition and Infection Study, HEU = HIV-exposed, uninfected, HUU = HIV-unexposed, uninfected

Figure 1 shows the flow diagram showing numbers of children in the original studies, numbers we were able to follow up at school age, and their HIV exposure status. A larger proportion (41 %) of the original CIGNIS cohort than of the original BFPH cohort (15 %) were followed up. Proportions of HIV-exposed children differed between the two cohorts because of the original study designs. HIV status of all but 3 HIV-exposed CIGNIS children was known; those 3 had missed the final 18 month project visit but came for follow-up. HIV status of only 7 of 32 HIV-exposed BFPH children was known; all were HIV-negative. The main analyses included 111 HEU children, that is, 32 from BFPH and 79 from CIGNIS, who were not known to be HIV-infected. Supplementary analyses were restricted only to the 83 children, 7 from BFPH and 76 from CIGNIS, who were confirmed HIV-negative. The 279 HIV-unexposed children, 32 from BFPH and 247 from CIGNIS, served as controls for both analyses.

Table 1 presents sociodemographic data divided by cohort and by HIV-exposure. Children from the CIGNIS cohort were of higher socioeconomic status than those from the BFPH cohort in terms of both parents’ education and employment status as well as the asset index. HEU children were slightly older than HUU children which reflects the larger proportion of HIV-exposed children in the BFPH cohort. Birth weight of HEU children was lower than that of HUU children. More HEU children had mothers who were widowed or divorced. HEU children were from families of lower socioeconomic status and parental education and employment which may result from several factors: more HEU children in the lower socioeconomic BFPH cohort, more divorced or widowed mothers, and possibly the effect of HIV-related illness on family income and expenditure.

**Table 1:** Characteristics of children at follow-up^a, b, c^

	BFPH cohort	CIGNIS cohort	HUU	HEU
N	64	326	279	111
Sex (# (%) male	30 (47 %)	146 (45 %)	131 (47 %)	45 (41 %)
Age (years) ^d^	11.6 (SD 0.7)	7.5 (SD 0.7)	8.0 (SD 1.5)	8.6 (SD 2.0)
Birth weight (kg)^d^	3.05 (SD 0.44)	3.03 (SD 0.51)	3.07 (SD 0.50)	2.94 (SD 0.49)
Marital status^d^				
Married	39 (61 %)	245 (75 %)	219 (79 %)	65 (59 %)
Widowed	10 (16 %)	13 (4 %)	8 (3 %)	15 (14 %)
Divorced	2 (3 %)	19 (6 %)	15 (5 %)	6 (5 %)
Single	9 (14 %)	35 (11 %)	33 (12 %)	11 (10 %)
Other/unknown	4 (6 %)	13 (4 %)	3 (1 %)	14 (13 %)
Mother’s education^d^				
Primary or less	40 (63 %)	102 (31 %)	94 (34 %)	48 (43 %)
Secondary	13 (20 %)	68 (21 %)	53 (19 %)	28 (25 %)
College/university	7 (11 %)	149 (46 %)	130 (47 %)	26 (23 %)
Missing	4 (6 %)	6 (2 %)	1 (0.4 %)	9 (8 %)
Father’s education^d, e^				
Primary or less	11 (17 %)	44 (14 %)	41 (15 %)	14 (13 %)
Secondary	25 (39 %)	84 (26 %)	63 (23 %)	46 (41 %)
College/university	10 (16 %)	168 (52 %)	151 (54 %)	27 (24 %)
Missing	18 (28 %)	29 (9 %)	23 (8 %)	24 (23 %)
Mother’s occupation^d, e^				
Employed	36 (56 %)	238 (73 %)	202 (73 %)	72 (65 %)
Housewife	18 (28 %)	56 (17 %)	54 (19 %)	20 (18 %)
Unemployed	6 (9 %)	24 (7 %)	21 (8 %)	9 (8 %)
Not applicable or unknown	4 (6 %)	7 (2 %)	1 (0.4 %)	10 (9 %)
Father’s occupation^d, e^				
Employed	42 (66 %)	283 (87 %)	242 (87 %)	83 (75 %)
Unemployed	3 (5 %)	10 (3 %)	10 (4 %)	3 (3 %)
Not applicable or unknown	18 (28 %)	28 (9 %)	26 (9 %)	25 (22 %)
Asset index tertiles^d, e^				
Low	31 (48 %)	99 (30 %)	78 (28 %)	52 (47 %)
Medium	24 (38 %)	109 (33 %)	99 (35 %)	36 (32 %)
High	9 (14 %)	118 (36 %)	104 (37 %)	23 (21 %)

^a^Defined inclusively as children either known negative or untested

^b^ *HEU* HIV-exposed, uninfected, *HUU* HIV-unexposed, uninfected

^c^Values are mean (SD) or number (%)

^d^Different between BFPH and CIGNIS, *P* < 0.05; t-tests used for age and birth weight, chi-square test for other variables

^e^Different between HUU and HEU, *P* < 0.05, by chi-square test

### Anthropometry and body composition

Table 2 shows anthropometric and body composition data for HUU children, HEU children not known to be HIV-positive, and HEU children confirmed negative. Because two cohorts of differing ages and proportion HIV-exposed are involved in the different groups, analyses of the effect of HIV exposure controlled for age (Table 3). Sex is also controlled for in the primary analyses. Z scores were not controlled for age or sex since they already account for these. In the age and sex-adjusted analyses, MUAC, hip circumference and percent fat overall and in trunk, legs and arms were significantly lower among HEU than HUU children with borderline lower BMI, thigh circumference and subscapular skinfold. Controlling for socioeconomic variables which differed between HEU and HUU children (mother’s marital status, maternal and paternal education, maternal and paternal occupation, and asset index) resulted in no significant anthropometric and body composition differences (Table 3). Similar results were seen if the HEU group was restricted to only children known to be HIV-negative (see Additional file 1: Table S1) or to only the CIGNIS children, *i.e.* the larger group and with better follow-up (results not shown). These results suggest that lower socioeconomic status was the main reason for lower anthropometric variables in the HEU children. Categorical analyses of anthropometric Z scores according to usual cut-offs similarly showed no differences (data not shown) except it is worth noting that all 16 children who were overweight (BMI Z score > 2) were HUU.

**Table 2:** Anthropometry and body composition of HEU and HUU children at follow-up ^a^

	HUU	HEU not confirmed HIV-positive	HEU confirmed HIV-negative
	Mean (SD)	Mean (SD)	Mean (SD)
	*N* = 279	*N* = 111	*N* = 83
Raw measures			
Weight (kg)	25.5 (7.6)	26.8 (8.9)	23.6 (5.8)
Height (cm)	125.0 (9.7)	128.2 (12.1)	123.6 (9.0)
BMI (kg/m^2^)	16.0 (2.7)	15.9 (2.5)	15.2 (2.0)
Waist circumference (cm)	55.5 (6.6)	56.0 (6.1)	54.2 (4.5)
Hip circumference (cm)	65.7 (8.7)	66.3 (9.2)	63.3 (6.9)
Thigh circumference (cm)	35.7 (5.2)	35.9 (5.5)	34.4 (4.2)
MUAC (cm)	18.7 (3.2)	18.7 (2.9)	17.8 (2.1)
Triceps skinfold (mm)	8.6 (3.8)	8.5 (3.4)	8.0 (2.6)
Subscapular skinfold (mm)	7.2 (3.5)	7.1 (3.0)	6.7 (2.6)
Z scores			
Height-for-age	−0.34 (0.93)	−0.39 (0.92)	−0.36 (0.89)
BMI-for age	−0.12 (1.20)	−0.26 (1.00)	−0.36 (0.98)
Bioelectrical impedance^b^			
Total fat percent	20.8 (4.9)	19.9 (3.9)	19.7 (3.6)
Trunk fat percent	14.9 (4.8)	14.0 (3.7)	13.8 (3.5)
Leg fat percent	29.6 (5.0)	28.5 (4.6)	28.7 (4.0)
Arm fat percent	31.1 (4.8)	30.0 (3.8)	30.4 (3.6)

^a^ *BMI* body mass index, *MUAC* mid-upper arm circumference, *HEU* HIV-exposed, uninfected, *HUU* HIV-unexposed, uninfected

^b^Bioelectrical impedance results missing from 14 HUU children and 2 HEU children because they were less than 7 years

**Table 3:** Associations of early HIV exposure with anthropometry and body composition – HUU children and all HEU children not confirmed HIV-positive^a,b^

	Adjusted for age and sex^c^ (coefficient, 95 % CI)	*P*	Fully adjusted^d^ (coefficient, 95 % CI)	*P*
Raw measures (*n* = 388)				
Weight (kg)	−0.93 (−2.15, 0.29)	0.13	−0.03 (−1.20, 1.15)	0.96
Height (cm)	−0.27 (−1.48, 0.94)	0.66	0.46 (−0.74, 1.67)	0.45
BMI (kg/m^2^)	−0.53 (−1.07, 0.01)	0.055	−0.19 (−0.71, 0.33)	0.48
Waist circumference (cm)	−0.77 (−2.03, 0.49)	0.23	−0.10 (−1.36, 1.15)	0.87
Hip circumference (cm)	−1.74 (−3.24, −0.24)	0.023	−0.56 (−1.98, 0.86)	0.44
Thigh circumference (cm)	−0.95 (−1.94, 0.04)	0.06	−0.31 (−1.27, 0.66)	0.53
MUAC (cm)	−0.63 (−1.23, −0.04)	0.037	−0.21 (−0.78, 0.37)	0.48
Triceps skinfold (mm)	−0.57 (−1.35, 0.21)	0.15	−0.02 (−0.76, 0.73)	0.97
Subscapular skinfold (mm)	−0.61 (−1.31, 0.10)	0.09	−0.15 (−0.83, 0.53)	0.66
Z scores (*n* = 388)				
Height-for-age	−0.03 (−0.24, 0.17)	0.75	0.09 (−0.11, 0.30)	0.38
BMI-for age	−0.18 (−0.44, 0.08)	0.18	−0.03 (−0.28, 0.22)	0.81
Bioelectrical impedance^b^ (*n* = 373)				
Total fat percent	−1.21 (−2.22, −0.21)	0.018	−0.64 (−1.62, 0.34)	0.20
Trunk fat percent	−1.06 (−2.07, −0.05)	0.039	−0.56 (−1.56, 0.43)	0.27
Leg fat percent	−1.29 (−2.31, −0.28)	0.013	−0.73 (−1.72, 0.26)	0.15
Arm fat percent	−1.16 (−2.15, −0.18)	0.021	−0.68 (−1.65, 0.29)	0.17

^a^ *BMI* body mass index, *CI* confidence interval, *MUAC* mid-upper arm circumference, *HEU* HIV-exposed, uninfected, *HUU* HIV-unexposed, uninfected

^b^Coefficients are differences between HEU and HUU controls

^c^Adjusted for age and sex for raw measures and bioelectrical impedance but not for Z scores

^d^Adjusted for age, sex, mother’s marital status, mother’s education, father’s education, mother’s occupation, father’s occupation and asset index tertile; Z scores not adjusted for age and sex

We examined baseline sociodemographic characteristics which differed between children who were or were not later followed up. For the BFPH cohort the only difference was that among children followed up, more fathers had been unemployed when their mothers were pregnant and their mothers weighed more at recruitment during pregnancy. For the CIGNIS cohort, among children followed up compared to those not followed, more of their mothers (34 % vs 25 %) had college or university education and more families had higher categories of an asset index. In both cohorts there was no difference in early anthropometric measures between those followed up and not (data not shown). Since the socioeconomic variables which differed between those followed up and not were similar to those which were already controlled for in adjusted analyses, we did not conduct further analyses controlling for missing at follow-up.

### Clinical and biochemical outcomes

There was no difference between HEU and HUU children in reported referral to clinic within the past month or hospital within the past year. However, more HEU than HUU children were reported to be ill at the time of the follow-up visit (50 % versus 37 %). The most common infections diagnosed by clinical examination were upper respiratory tract infections and worm infestations, 40–45 % of children for each, and skin conditions, 8 %, with no difference between HEU and HUU children. More HEU than HUU children (41 % versus 30 %) were prescribed medication following examination; the most common medications were anti-helminths, antibiotics, skin creams, and pain killers. Blood pressure was marginally higher among HEU children (Table 4) but this difference was not significant in the restricted analysis of only children confirmed HIV-negative (see Additional file 2: Table S2). Biochemical results were remarkably similar between HUU and HEU children and, unlike for anthropometry, control for socioeconomic variables which differed between groups had virtually no effect on point estimates of biochemical differences. Controlling for the time and nature of the most recent drink, snack or meal, since not all children were fasting, did not change the result.

**Table 4:** Associations between early HIV-exposure and biochemical and school report results^a, b^

	HUU		HEU		Unadjusted analysis		Adjusted analysis^c^	
	Mean (SD)	N	Mean (SD)	N	B (95 % CI)	P	B (95 % CI)	P
Blood pressure								
Systolic (mmHg)	89 (9)	275	92 (11)	110	2.7 0.7, 4.7)	0.009	1.7 (−0.2, 3.5)	0.07
Diastolic (mmHg)	58 (8)	275	60 (9)	110	1.7 (0.1, 3.6)	0.07	1.4 (−0.4, 3.2)	0.13
Blood biochemistry								
CRP (mg/L)^d^	1.40 (1.15, 1.70)	209	1.49 (1.07, 2.06)	91	0.06 (−0.31, 0.43)	0.74	0.15 (−0.23, 0.53)	0.44
Cholesterol (mg/dl)	159 (58)	260	152 (67)	106	3 (−10, 16)	0.67	0 (−14, 13)	1.0
Haemoglobin (g/L)	128 (12)	275	128 (14)	109	−0.3 (−3, 2)	0.83	−0.6 (−3, 2)	0.70
HbA1c (%)^d^	5.44 (5.37, 5.51)	276	5.38 (5.29, 5.48)	110	−0.01 (−0.03, 0.01)	0.37	0 (−0.03, 0.02)	0.81
Glucose (mmol/L)	5.4 (1.2)	85	5.4 (0.7)	33	0 (−0.43, 0.43)	1.0	−0.03 (−0.49, 0.44)	0.90
School grades								
Math (%)	77 (19)	98	65 (24)	27	−12.2 (−21.9, −3.6)	0.006	−9.6 (−18.3, −1.0)	0.029
English (%)	79 (18)	97	75 (24)	26	−3.2 (−11.8, 5.4)	0.46	−0.6 (−9.2, 7.9)	0.88

^a^ Includes all HEU children not known to be HIV-positive

^b^ *CRP* C-reactive protein, CI-confidence interval, *HEU* HIV-exposed, uninfected, *HUU* HIV-unexposed, uninfected

^c^Adjusted for age, sex, mother’s marital status, mother’s education, father’s education, mother’s occupation, father’s occupation and asset index tertile

^d^Geometric means and 95 % CIs are presented for HUU and HEU results for ease of interpretation but regression coefficients are from the analyses using data transformed to natural logs

### School reports

All but 8 children (2 %) attended school but only 125 (33 %) of mothers brought their child’s most recent school report to the clinic visit as requested; HEU and HUU groups did not differ in these. Children with and without school reports were very similar in terms of sociodemographic variables except for maternal occupation: 72 % of mothers who did not bring reports, compared with 66 % who did, were employed outside the home. Children in the CIGNIS cohort scored significantly higher in both English and math than those in the BFPH cohort (data not presented), likely because the courses and expectations are very different for the younger and older children. There were no differences in English grades between groups but HEU children had lower grades in math than HUU children and this difference was maintained after controlling for sociodemographic variables. Controlling for the original study in the analysis did not change these results. However, the difference in math scores was not seen in the restricted analysis of only children confirmed HIV-negative, possibly because this excluded most of the older, BFPH, children.

## Discussion

Our results contribute to the extremely limited data on long term follow-up of HEU African children in comparison with appropriate HUU controls. School-aged HEU children had lower values of most anthropometric variables than HUU children but all significant differences vanished after control for socioeconomic variables differing between the groups of children. This suggests that, in spite of clear evidence of early nutritional or other stresses as indicated by lower birth weight and early growth, in both BFPH and CIGNIS cohorts as well as in other African cohorts [1, 12] and immune abnormalities [8], the main factors impairing growth in HEU by school-age are socioeconomic. We detected little or no evidence of functional differences between HEU and HUU children in terms of markers of acute or chronic diseases or school performance, other than more children reporting illness or requiring medication at the follow-up visit, slightly higher systolic blood pressure, and lower math grades in the larger HEU cohort of all children not known to be HIV-infected. Increased reporting of illness could have reflected real illness but could also have been a reporting bias in HIV-affected families where illness is common; we note that most clinical conditions were mild and there were no differences in the inflammatory marker, CRP, between HEU and HUU children suggesting lack of clinically important differences in illness.

Adequately controlled studies of the effect of maternal HIV exposure on child health outcomes are rare, in part because of the difficulties of distinguishing between HIV exposure itself and socioeconomic factors associated with parental HIV infection. In North America and Europe, most HIV-exposed children come from populations very different in terms of ethnic composition or socioeconomic status and it can be hard to control for this sufficiently to determine the effects of HIV exposure [23, 24]. Most research in Africa conducted around the time of the BFPH and CIGNIS studies focused on PMTCT interventions and thus did not recruit HIV-negative mothers as controls. Comparison of growth of HEU children with children from national surveys is usually inappropriate since participants in research studies, particularly in low-income countries where even controls, for ethical reasons, often receive more medical and other care than is available outside the research setting, are likely to have better health outcomes than community controls. We note that participating in the control arm of the CIGNIS trial resulted in a halving of the stunting rate compared with children from the same clinic who reached the recruitment age of 6 months just before or after the CIGNIS recruitment period and were thus ineligible [10]. We found more socioeconomic and demographic differences between HEU and HUU in both BFPH and CIGNIS cohorts at follow-up than at recruitment. This may reflect the changing economic situation in Lusaka, increasing illness or death of parents as their HIV progresses, and the biased follow-up in relation to socioeconomic status. Recent studies of HEU children also need to account for ART exposure which is associated with some fetal growth and health problems [1] but, at the time BFPH and CIGNIS children were born, the only drug available locally in Lusaka for PMTCT was perinatal single dose nevirapine.

In addition to changes in ART availability, infant feeding recommendations for HIV-infected mothers have changed rapidly over the past couple of decades. The BFPH and CIGNIS studies were done at different times over this period of rapid change in recommendations and practices. In BFPH there was little difference in duration of either exclusive or any breastfeeding between HIV-infected and uninfected women and both groups breastfed until the children were about 18 months old. In contrast, many HIV-infected CIGNIS mothers did not initiate breastfeeding and those who did start tended to stop within the first year [18]. Although decreased breastfeeding could have contributed to differences between HEU and HUU children, we note that the math score differences were seen mainly for the BFPH children where breastfeeding duration did not differ between groups.

A number of studies have investigated cognitive development among HEU African children, most focussing on children under 5 years and most from the pre-ART era. At 18 months of age the HIV-exposed CIGNIS children had lower mental and motor development than the unexposed children [14]. HIV-uninfected Congolese toddlers with seriously ill or dead HIV-infected parents had delayed motor development and language expression but analyses did not control for socioeconomic differences between HEU and HUU groups [25]. There were no differences in mental or motor development between HEU and HUU Ugandan children aged 6–24 months [26]. In a population participating in an open-label ART trial, Zambian HEU toddlers did not differ in cognitive development from community controls but there may have been a benefit to the HEU group from belonging to a research study [27]. HEU young Ugandan children who had similar home environments and caretaker interactions as HUU controls did not differ in mental or motor development from the controls [28]. Some of these children were followed up to school age at which point there were also no cognitive differences between HEU and HUU children [29]. This study also found no impairment among HIV-infected children which may have been because only a minority survived beyond age 4 years. Although school grades, as used in our study, reflect more than simply cognitive development, they have the merit of being associated with future education and employment. Our finding of lower math scores could be real but could also be a chance finding resulting from multiple comparisons. Overall it appears that there are minimal cognitive impairments among HEU children other than those resulting from poor parental health and child interactions or poor socioeconomic conditions. However, the high prevalence of early cytomegalovirus infection which is associated with early developmental delay among HIV-exposed children [7] and can lead to hearing loss [30] could impair learning in school. Therefore, the development of HEU children should be monitored in clinics or schools to permit remedial help or hearing support as needed.

Strengths of our study are its fairly large sample size and the careful anthropometric, body composition and other measurements conducted by an experienced clinical team. A major limitation is the large and biased loss to follow-up between the original BFPH and CIGNIS studies and measurements when the children were school-aged plus the further loss in school data because of missing reports. We controlled for some indicators of socioeconomic status but unmeasured confounding remains a concern. It is possible that some children not followed had died and, since early childhood death is more common among HEU children [4], the group at follow-up may represent the healthiest children, *i.e.* there could be a survivor bias limiting any differences due to early HIV exposure. Our definitions of HEU and HUU were not the strictest but we believe are reasonable. For our main analyses we included as HEU all HIV-exposed children not known to be HIV-infected. It was mostly BFPH children who had not been tested and, with a mean age over 11 years, we expect most infected children would be showing symptoms and yet none of the untested children did. Secondly, our restricted analysis including only children known to be HIV-negative found very similar results to the more inclusive analysis. An exception was the difference in math scores which was seen only when the untested children, mostly from BFPH, were included. It is possible this is a true difference because the types of school courses, the skills involved, and the style of reports differed considerably for the younger CIGNIS children and the older BFPH children. There were also likely differences between recording of grades by different teachers and in different schools; our sample size, further limited by missing reports, was not sufficient to control for this which would likely have decreased the variability and perhaps thus increased chances of detecting differences. Finally, since we were interested in early HIV exposure, we included as HUU 4 BFPH children and 7 CIGNIS children whose mothers had later seroconverted. These families may have experienced the poorer socioeconomic status of HIV-affected families; however, this is unlikely to have had much effect on our results since including these children as HUU would have biased the differences towards null which they mostly were already in adjusted analyses.

## Conclusions

HIV-uninfected children exposed to maternal HIV in early life exhibited lower anthropometric scores at school age than did HIV-unexposed children but this appeared to be mainly due to the lower socioeconomic status of HIV-infected families. Measures of biochemical markers of acute or chronic diseases did not differ between HEU and HUU children. Effects on school performance require further research. Since sociodemographic effects were important contributors to poorer growth of HEU children, it will be important to investigate HEU children in different African populations in order to provide information beyond the specific Lusaka context.

### Availability of supporting data

Requests for data access for scientific research abiding by the principles under which the data were collected should be made to S Filteau.

## Additional files

Additional file 1: Table S1. Associations of early HIV exposure with anthropometry and body composition – restricted analysis with confirmed HIV-negative children only. Additional file 2: Table S2. Blood pressure, biochemical data and school report results for children confirmed HIV-negative.

## Abbreviations

ART: Antiretroviral therapy
BMI: Body mass index
HEU: HIV-exposed, uninfected
HUU: HIV-unexposed, uninfected
MUAC: Mid-upper-arm circumference
PMTCT: Prevention of mother-to-child HIV transmission
SD: Standard deviation
SES: Socioeconomic status
