# Supplementary material for: Growth and health outcomes at school age in HIV-exposed, uninfected Zambian children: follow-up of two cohorts studied in infancy
Source: BMC Pediatr. 2015 Jun 6;15:66. doi: 10.1186/s12887-015-0386-8 (PMC4458018; doi:10.1186/s12887-015-0386-8)
Supplement: Additional file 1: Table S1. — Associations of early HIV exposure with anthropometry and body composition – restricted analysis with confirmed HIV-negative children only. [file 12887_2015_386_MOESM1_ESM.pdf]

Supplementary Table 1. Associations of early HIV exposure with anthropometry and body composition – restricted analysis with confirmed HIV-negative children only[1,2]

	Adjusted for age and sex[3] (coefficient, 95% CI)	P	Fully adjusted[4] (coefficient, 95% CI)	P
Raw measures (n=360)				
Weight (kg)	-1.17 (-2.45, 0.12)	0.07	-0.37 (-1.61, 0.88)	0.56
Height (cm)	-0.21 (-1.52, 1.09)	0.75	0.50 (-0.81, 1.80)	0.46
BMI (kg/m^2)	-0.65 (-1.24, -0.06)	0.031	-0.34 (-0.91, 0.23)	0.24
Waist circumference (cm)	-0.86 (-2.22, 0.51)	0.22	-0.24 (-1.60, 1.11)	0.72
Hip circumference (cm)	-1.86 (-3.49, -0.23)	0.025	-0.79 (-2.32, 0.81)	0.32
Thigh circumference (cm)	-1.03 (-2.09, 0.04)	0.06	-0.41 (-1.45, 0.63)	0.44
MUAC (cm)	-0.75 (-1.40, -0.10)	0.025	-0.35 (-0.99, 0.28)	0.28
Triceps skinfold (mm)	-0.64 (-1.48, 0.20)	0.13	-0.11 (-0.91, 0.69)	0.79
Subscapular skinfold (mm)	-0.46 (-1.22, 0.30)	0.23	-0.04 (-0.78, 0.70)	0.92
Z scores (n=360)				
Height-for-age	-0.03 (-0.26, 0.20)	0.79	0.09 (-0.13, 0.32)	0.42
BMI-for age	-0.24 (-0.52, 0.05)	0.11	-0.10 (-0.38, 0.18)	0.49
Bioelectrical impedance (n=345)				
Total fat percent	-1.25 (-2.36, -0.13)	0.029	-0.70 (-1.79, 0.39)	0.21
Trunk fat percent	-1.14 (-2.27, 0.02)	0.047	-0.69 (-1.80, 0.42)	0.22
Leg fat percent	-1.28 (-2.39, 0.16)	0.025	-0.73 (-1.82, 0.36)	0.19
Arm fat percent	-1.02 (-2.12, 0.08)	0.07	-0.54 (-1.62, 0.54)	0.32

[1]BMI=body mass index, CI-confidence interval, MUAC=mid-upper arm circumference, HEU=HIV-exposed, uninfected, HUU=HIV-unexposed, uninfected
[2]Coefficients are differences between HEU and HUU controls
[3]Adjusted for age and sex for raw measures and bioelectrical impedance but not for Z scores
[4]Adjusted for age, sex, mother's marital status, mother's education, father's education, mother's occupation, father's occupation and asset index tertile; Z scores not adjusted for age and sex
