# Supplementary material for: Growth and health outcomes at school age in HIV-exposed, uninfected Zambian children: follow-up of two cohorts studied in infancy
Source: BMC Pediatr. 2015 Jun 6;15:66. doi: 10.1186/s12887-015-0386-8 (PMC4458018; doi:10.1186/s12887-015-0386-8)
Supplement: Additional file 2: Table S2. — Blood pressure, biochemical data and school report results for children confirmed HIV-negative. [file 12887_2015_386_MOESM2_ESM.pdf]

Supplementary Table 2. Blood pressure, biochemical data and school report results for children confirmed HIV-negative[1]

	HUU		HEU		Unadjusted analysis		Adjusted analysis[2]	
	Mean (SD)	N	Mean (SD)	N	B (95% CI)	P	B (95% CI)	P
<u>Blood pressure</u>								
Systolic (mmHg)	89 (9)	275	89 (10)	82	0.2 (-2.0, 2.4)	0.85	1.4 (-0.6, 3.4)	0.18
Diastolic (mmHg)	58 (8)	275	58 (9)	82	-0.2 (-2.3, 1.8)	0.83	1.0 (-1.0, 3.0)	0.32
<u>Blood biochemistry</u>								
CRP (mg/L)[3]	1.40 (1.15, 1.70)	209	1.66 (1.13, 2.45)	66	0.17 (-0.24, 0.58)	0.42	0.14 (-0.28, 0.57)	0.51
Cholesterol (mg/dl)	159 (58)	260	166 (52)	78	7 (-8, 21)	0.36	6 (-9, 21)	0.42
Haemoglobin (g/L)	128 (12)	275	126 (14)	81	-2 (-5, 2)	0.32	-1 (-4, 2)	0.52
HbA1c (%)[3]	5.44 (5.37, 5.51)	276	5.41 (5.30, 5.52)	82	-0.01 (-0.03, 0.02)	0.67	0 (-0.03, 0.02)	0.77
Glucose (mmol/L)	5.4 (1.2)	85	5.5 (0.6)	27	0.02 (-0.45, 0.50)	0.92	0.01 (-0.50, 0.53)	0.96
<u>School grades</u>								
Math (%)	77 (19)	98	70 (20)	21	-6.5 (-15.7, 2.7)	0.16	-4.3 (-13.4, 4.7)	0.35
English (%)	79 (18)	97	82 (18)	21	2.9 (-5.8, 11.7)	0.51	5.2 (-3.6, 14.0)	0.25

[1] CRP=C-reactive protein, CI-confidence interval, HEU=HIV-exposed, uninfected, HUU=HIV-unexposed, uninfected

[2] Adjusted for age, sex, mother's marital status, mother's education, father's education, mother's occupation, father's occupation and asset index tertile

[3] Geometric means and 95% CIs are presented for HUU and HEU results for ease of interpretaion but regression coefficients are from the analyses using data transformed to natural logs.
